# Age‐related neuromuscular changes affecting human vastus lateralis

**DOI:** 10.1113/JP271087

**Published:** 2015-12-15

**Authors:** M. Piasecki, A. Ireland, D. Stashuk, A. Hamilton‐Wright, D. A. Jones, J. S. McPhee

**Affiliations:** ^1^ School of Healthcare ScienceManchester Metropolitan UniversityManchesterUK; ^2^ Department of Systems Design EngineeringUniversity of Waterloo, WaterlooOntarioCanada; ^3^ Mathematics and Computer ScienceMount Allison University, SackvilleNew BrunswickCanada

## Abstract

**Key points:**

- Skeletal muscle size and strength decline in older age.
- The vastus lateralis, a large thigh muscle, undergoes extensive neuromuscular remodelling in healthy ageing, as characterized by a loss of motor neurons, enlargement of surviving motor units and instability of neuromuscular junction transmission.
- The loss of motor axons and changes to motor unit potential transmission precede a clinically‐relevant loss of muscle mass and function.

**Abstract:**

The anterior thigh muscles are particularly susceptible to muscle loss and weakness during ageing, although how this is associated with changes to neuromuscular structure and function in terms of motor unit (MU) number, size and MU potential (MUP) stability remains unclear. Intramuscular (I.M.) and surface electromyographic signals were recorded from the vastus lateralis (VL) during voluntary contractions held at 25% maximal knee extensor strength in 22 young (mean ± SD, 25.3 ± 4.8 years) and 20 physically active older men (71.4 ± 6.2 years). MUP size, firing rates, phases, turns and near fibre (NF) jiggle were determined and MU number estimates (MUNEs) were made by comparing average surface MUP with maximal electrically‐evoked compound muscle action potentials. Quadriceps cross‐sectional area was measured by magnetic resonance imaging. In total, 379 individual MUs were sampled in younger men and 346 in older men. Compared to the MU in younger participants, those in older participants had 8% lower firing rates and larger MUP size (+25%), as well as increased complexity, as indicated by phases (+13%), turns (+20%) and NF jiggle (+11%) (all *P* < 0.0005). The MUNE values (derived from the area of muscle in range of the surface‐electrode) in older participants were ∼70% of those in the young (*P* < 0.05). Taking into consideration the 30% smaller cross‐sectional area of the VL, the total number of MUs in the older muscles was between 50% and 60% lower compared to in young muscles (*P* < 0.0005). A large portion of the VL MU pool is lost in older men and those recruited during moderate intensity contractions were enlarged and less stable. These MU changes were evident before clinically relevant changes to muscle function were apparent; nevertheless, the changes in MU number and size are probably a prelude to future movement problems.

## Key points

- Skeletal muscle size and strength decline in older age.
- The vastus lateralis, a large thigh muscle, undergoes extensive neuromuscular remodelling in healthy ageing, as characterized by a loss of motor neurons, enlargement of surviving motor units and instability of neuromuscular junction transmission.
- The loss of motor axons and changes to motor unit potential transmission precede a clinically‐relevant loss of muscle mass and function.

**Abbreviations**
ALM: appendicular lean mass
BMI: body mass index
CMAP: compound muscle action potential
CSA: cross‐sectional area
EMG: electromyography
FDI: first dorsal interosseous
iEMG: intramuscular electromyography
IQR: interquartile range
MVC: maximum voluntary contraction
MRI: magnetic resonance imaging
MU: motor unit
MUP: motor unit potential
MUPT: motor unit potential train
NF: near fibre
NMJ: neuromuscular junction
sEMG: surface electromyography
sMUP: surface motor unit potential
SPPB: Short Physical Performance Battery
VL: vastus lateralis
VM: vastus medialis

## Introduction

A decrease in mobility and a loss of independence are common experiences of ageing and, although many factors contribute to this, a loss of muscle mass and function is central to the process. Muscle can only function in tandem with the nervous system. Neural control of muscle is organized through motor units (MU), which consist of a single alpha motor neuron and all of the muscle fibres that it innervates (Sherrington, 1925) and, in large limb muscles, there are many thousands of MUs (Tomlinson & Irving, 1977). When considering the reasons for age‐related loss of muscle mass and function and the factors that might influence any changes, for better or worse, it is clearly essential to take into account any alterations in the organization and control of the MUs.

Post mortem anatomical studies have shown that, compared to young adults, those aged ∼75 years have ∼30% fewer motor neurons supplying the muscles of the lower limbs (Kawamura *et al*. 1977; Tomlinson & Irving, 1977; Mittal & Logmani, 1987), whereas Lexell *et al*. (1988) reported 40% fewer fibres in the vastus lateralis (VL) of older quadriceps muscles. Such studies clearly indicate the probable importance of neurological changes affecting muscle function, although methods that can be used with living human subjects are required for more detailed investigations.

Campbell *et al*. (1973) recorded evoked potentials and compared these with maximum M‐waves to estimate that there are ∼50% fewer MUs in the extensor digitorum brevis of people aged >60 years. However, there is some uncertainty about this technique; the main problem being ‘alternation’, occurring when MUs have similar thresholds (McComas *et al*. 1993). These complications are largely overcome by intramuscular (I.M.) recordings obtained from single MUs during voluntary contractions and using ‘spike triggered averaging’ to extract MU potentials (MUP) from the surface recorded signal Brown *et al*. (1988). Subsequent developments in signal decomposition enhancement have removed most of the subjective interpretations of complex electromyography (EMG) signals (Gooch *et al*. 2014). By comparing the average size (either area or amplitude) of the surface MUP (sMUP) with that of a compound muscle action potential elicited by supramaximal stimulation of the motor nerve, it is possible to estimate the number of MU in the muscle, providing a MU number estimate (MUNE) value.

MUNE values have shown older people to have fewer MUs in peripheral limb muscles, such as the tibialis anterior and extensor digitorum brevis (Galea, 1996; McNeil *et al*. 2005; Power *et al*. 2010; Hourigan *et al*. 2015). However, no MUNE data are available for the anterior thigh muscles, which provide the power needed for daily locomotion, such as stair negotiation or rising from a seated position. It is important to obtain MU data on the thigh muscles because not only are they functionally relevant, but also they appear to be particularly susceptible to the effects of ageing. For example, the leg muscles undergo greater loss of mass with ageing than those in the upper body (Janssen *et al*. 2000) and even the muscles of the legs do not deteriorate equally during ageing (Abe *et al*. 2014). Cross‐sectional studies show that, with respect to size, the hamstrings, triceps surae and tibialis anterior muscles are relatively well preserved and the quadriceps are most affected by age‐related losses (Abe *et al*. 2011; Maden‐Wilkinson *et al*. 2013). Furthermore, a longitudinal study of 12 older people investigated at ∼71 years and again at ∼80 years found a reduction in the cross‐sectional area (CSA) of the anterior thigh muscles, although there was no such reduction in the posterior thigh muscles (Frontera *et al*. 2008). Taken together, these studies indicate that the quadriceps are more susceptible to age‐related atrophy and are therefore of particular interest in studies of neuromuscular decline.

In addition to MUNE values, i.m. EMG recordings provide details of MU discharge rates, as well as shape and stability of the MUPs, which indicate MU remodelling and the stability of transmission at the neuromuscular junction (NMJ). Specifically, these are demonstrated in terms of MUP area, number of turns, number of phases and jiggle, with the latter statistic reflecting variations in individual MUP shapes (Stashuk, 1999 *a*, *b*; Abdelmaseeh *et al*. 2014). A novel ‘near fibre’ (NF) method has recently been developed that allows examination of contributions only from fibres located very close to the recording needle electrode, thereby reducing artefact or attenuation as signals pass through muscle, fat and connective tissues (Allen *et al*. 2015).

Hourigan *et al*. (2015) recently identified larger MUPs and, for the first time in healthy ageing individuals, reported increased NF jiggle in the vastus medialis (VM) of nine older men compared to nine younger men, indicative of larger MUs as a result of re‐innervation and increased NMJ transmission variability (Stålberg & Sonoo, 1994). Ling *et al*. (2009) detected surface MUPs from i.m. recordings in the VM to show that older people recruited larger MUs compared to the young during low and moderate intensity contractions. However, MUNE values and muscle size were not obtained in either of these studies (Ling *et al*. 2009; Hourigan *et al*. 2015).

The present study aimed to apply the range of recently developed techniques for studying MU number and organization to the question of age‐related changes in the quadriceps muscle, comprising a muscle that is not only functionally important for mobility, but also particularly susceptible to the effects of ageing.

## Methods

### Ethical approval

The present study was approved by the University Research Ethics Committee and was conducted in accordance with the *Declaration of Helsinki*. All participants provided their written informed consent. Volunteers were included if they were male, aged between 18 and 35 years or 65 and 90 years and habitually physically active. Volunteers were excluded if they were sedentary or competing in sports at a regional level or above, had a recent history of bone fracture, or neuromuscular, metabolic or cardiovascular diseases. All older participants were asked to self‐rate the amount of time during which they had performed physical activities for health, fitness or leisure purposes (not including usual domestic activities) in the previous 7 days, and also whether the previous 7 days was representative of a ‘normal’ week.

### Anthropometry and general mobility

Body mass (kg) and height were measured and total body composition assessed by dual‐energy X‐ray absorptiometry (Lunar Prodigy Advance, version EnCore 10.50.086; GE Healthcare, Little Chalfont, UK) with the participant lying supine with legs and arms fully extended. Details of fat mass and lean mass of the whole body and of the body segments on the left and right sides were recorded. Sarcopenia (clinically‐relevant loss of muscle mass) was defined in accordance with the original criteria as appendicular lean mass divided by height‐squared (ALM h^–2^) (Baumgartner *et al*. 1998). A cut‐off of <6.76 ALM h^–2^ was used to identify older participants as sarcopenic, indicating a value below two SDs of the mean of a young, male European reference population (Bijlsma *et al*. 2013).

The sarcopenia definitions, which also include assessments of grip strength and poor mobility, were used additionally to characterize participants (Cruz‐Jentoft *et al*. 2010). Grip strength was assessed using a Jamar hand grip dynamometer (Patterson Medical, Warrenville, IL, USA). The right hand and left hand were tested separately, three times each, when the participant was standing with their arms extended by their side (McPhee *et al*. 2013). Weakness was defined as having a grip strength <32 kg, which is two SDs below the average of a young, male European reference population (Dodds *et al*. 2014). The Short Physical Performance Battery (SPPB) (Guralnik *et al*. 1994) was used to identify clinically‐relevant limitations with respect to balance, walking speed and the ability to stand from a seated position. A maximum of 12 points indicates no mobility limitations, whereas <8 points has been used to indicate sarcopenia or frailty (Bauer *et al*. 2015). Balance was assessed in three ways with increasing difficulty, and a maximum of 4 points could be gained. The participant was required to stand upright, with the arms beside their body for at least 10 s without needing support or taking a step, first with feet side‐by‐side as close together as possible (one point); second, with feet in a semi‐tandem position (one point) and, finally, with the feet fully tandem (two points for achieving 10 s, one point for achieving between 4–9.9 s). During tests of ‘normal’ gait speed, four points were awarded for completing the 4 m track in less than 4.82 s; three points for 4.82–6.2 s; two points for 6.21–8.7 s; one point for >8.71 s; and no points for failure to walk 4 m. The five‐times‐chair‐rise test required participants to stand‐up and sit‐down five times as quickly as possible. Four points were awarded for completing the task in <11.2 s; three points for 11.21–13.69 s; two points for 13.7–16.69 s; one point for >16.69 s; and no points for failure to complete the test.

### Knee extensor size and maximum voluntary isometric strength

Magnetic resonance imaging (MRI) was used to measure peak quadriceps and VL muscle anatomical CSAs using a T1‐weighted turbo 3‐D sequence on a 0.25‐T G‐Scan (Esaote, Genoe, Italy). Contiguous transverse‐plane slices of 6 mm thickness were collected. Images were analysed off‐line using Osirix imaging software (OsiriX Medical Imaging; OsiriX, Atlanta, GA, USA) and the slice with the highest quadriceps CSA and also the CSA of the VL at the mid‐belly motor point were recorded.

For assessments of knee extensor strength, participants were seated in a purpose built dynamometer with hip and knees flexed at ∼90 deg. The right leg was securely fastened to the force transducer, 30 cm below the centre of the knee joint (identified by palpation between the lateral femoral condyle and the tibio‐femoral contact point). After a standardized warm up, participants were instructed to perform a maximal isometric knee extension, with real‐time visual feedback and verbal encouragement from the investigators. This was repeated a further two times, with short rest intervals, and the highest value was taken as maximum voluntary contraction (MVC).

### Identifying the mid‐belly VL motor point

The VL motor point was identified as the site that produced the largest twitch after exploration along the VL longitudinal axis using low intensity percutaneous electrical stimulations (400 V, pulse width at 50 μS, current ∼8 mA using a Digitimer DS7A (Digitimer, Welwyn Garden City, UK) (Botter *et al*. 2011). The skin over the motor point was then prepared by shaving and cleansing with an alcohol swab.

### Set up for surface EMG (sEMG)

The active recording sEMG electrode was placed over the motor point (disposable self‐adhering Ag‐AgCl electrodes; 95mm^2^_;_ Ambu Neuroline, Baltorpbakken, Ballerup, Denmark). A reference electrode was placed over the patella tendon and a ground electrode placed over the patella (Ambu Neuroline Ground). Surface EMG signals were sampled at 10 kHz and bandpass filtered at 5 Hz to 5 kHz (1902 amplifier, Cambridge Electronics Design Ltd, Cambridge, UK).

EMG and force transducer signals were digitized (CED Micro 1401; Cambridge Electronic Design, Cambridge, UK) and displayed in real time using a computer interface running Spike2, version 8.01. Cambridge Electronic Design) and data were stored for off‐line analysis.

### Determining the maximal compound muscle action potential (CMAP)

The CMAP (or maximum M‐wave) was evoked by a manually triggered stimulator (model DS7A; Digitimer) using percutaneous stimulation (Medserve, Daventry, UK) of the femoral nerve (approximately halfway between the anterior superior iliac spine and the pubic tubercle, proximal to the groin crease but distal to the inguinal nerve) and a carbon‐rubber anode electrode (Dermatrode self‐adhering electrode, 5.08 cm in diameter; Farmadomo Linde Homecare Benelux Bv, Leiden, The Netherlands) placed over the skin overlying the gluteus muscle. The recording sEMG electrode was positioned to ensure the largest M‐wave with fastest rise time. The stimulator voltage was fixed at 400 V and the pulse width at 50 μS, and the current was then increased incrementally until the M‐wave amplitude plateaued. At this point, the current was increased again by 30 mA to ensure supramaximal stimulation (usually occurring between 100 and 150 mA). The CMAP was determined prior to insertion of the needle electrode.

### Set up for i.m. EMG

i.m. EMG signals were recorded using a 25 mm disposable concentric needle electrode with a recording area of 0.07 mm^2^ (Model N53153; Teca, Hawthorne, NY, USA) inserted diagonally at ∼60 deg close to the surface electrode to record from the muscle underneath the surface electrode. The iEMG shared the same ground electrode with the sEMG, which was placed over the patella. iEMG signals were sampled at 25 kHz and bandpass filtered at 10 Hz to 10 kHz (CED 1902 amplifier; Cambridge Electronics Design Ltd).

### Recording EMG during voluntary contractions

With the participant sitting fully relaxed, the skin immediately adjacent to the active sEMG electrode was pulled taut and a concentric needle was inserted to a depth of 1.5–2 cm. The participant performed a voluntary, low intensity knee extension and the needle was positioned to ensure that MUPs of sufficient sharpness (>5 kV s^2^) were detected. The participant then performed a voluntary contraction lasting 15 s, keeping as close as possible to a target line set at 25% MVC. Visual feedback was provided in real‐time. The concentric needle was then repositioned by combinations involving twisting the bevel by 180 deg and withdrawing the needle by ∼2–5 mm. The procedure of needle positioning, voluntary contraction and signal recording was repeated until a minimum of six recordings from spatially distinct regions at varying depths in the VL had been obtained. The participant rested for 15–30 s between contractions.

### Decomposition‐enhanced spike triggered averaging EMG analysis to extract MUPs, sMUPs and derive a MUNE value

DQEMG software was used to automatically identify MUPs, their corresponding sMUPs and to calculate MUNE values, as described and validated previously (Stashuk, 1999 *b*; Boe et al., 2004, 2006). All MUP and sMUP templates were visually inspected and their markers adjusted, where required, to correspond to the onset, onset of negative phase (sMUP only), end, and positive and negative peaks of the waveforms.

#### MUPs

**Figure 1:**
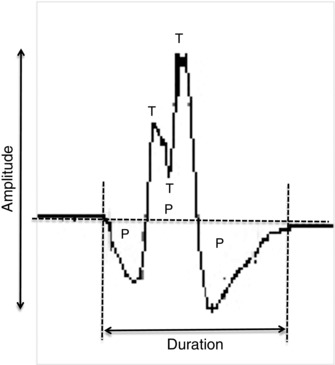
**A single MUP detected by iEMG** The MUP shows phases (P), turns (T), amplitude and duration. MUP area is calculated as the total area across its duration (i.e. between its onset and end).

Individual MUPs from motor unit potential trains (MUPTs) of the separate MUs were identified from the iEMG signal. MUPTs that were composed of MUPs from more than one MU or had fewer than 40 MUPs were excluded. MUP amplitude was measured from the maximal positive and negative peaks and the area was taken as the total area within the MUP duration (onset to end) (Fig. 1).

#### sMUPs

The corresponding sMUP was automatically identified for each MUP and visual inspection was used to reject any sMUPs if the onset did not occur within 10 ms of the MUP. The individual MUPs were used to ‘trigger’ the corresponding sMUP recorded by the sEMG. The sMUPs were then aligned and ensemble‐averaged to estimate an overall mean sMUP, which was taken as representative of the ‘size’ of the average MU recorded during the contraction at 25% MVC.

#### MUNE value

The MUNE value was derived from the ensemble‐averaged sMUP and CMAP using the original calculation (Brown *et al*. 1988; Stashuk, 1999 *b*). This was achieved in two ways. The first divided the negative peak area of the mean sMUP into the negative peak area of the CMAP; the second divided the negative peak amplitude of the mean sMUP into the negative peak amplitude of the CMAP. These are referred to as ‘MUNE area’ and ‘MUNE amplitude’, respectively. This decomposition‐enhanced spike‐triggered averaging provided by DQEMG is currently the standard technique for deriving a MUNE value (Gooch *et al*. 2014) and avoids the problems associated with multiple stimulation techniques where variable MU firings across MUs with similar stimulus intensity thresholds occur (Brown *et al*. 1988).

### MU discharge rates, complexity and NMJ transmission stability

The identified individual MUPs of a MUPT from the iEMG signal were used to assess the MU discharge rate, complexity and NMJ transmission stability. For discharge rate analysis, the MUPs within a MUPT were identified and expressed as a function of recording time (s^–1^, or Hz). The complexity of the MUs was assessed from the number of phases and turns of the template MUPs. The number of ‘phases’ of the template MUP was defined as the number of components above or below the baseline. A ‘turn’ was defined as a change in direction of the waveform of at least 25 μV (Fig. 1). Signal intensity was taken as the average, maximum number of peaks s^–1^ across 500 ms windows within the iEMG signal, and is a reflection of the level of muscle activity within the recording area of the needle electrode.

**Figure 2:**
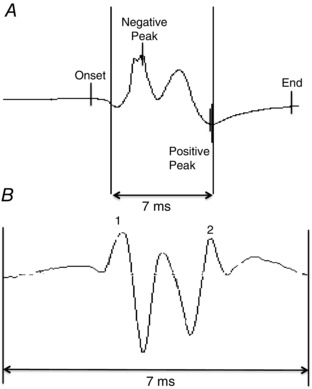
**A MUP and corresponding NF MUP detected by iEMG** A MUP is high pass filtered in DQEMG using a second‐order, low‐pass differentiator to generate a NF MUP. The MUP (*A*) shows onset, positive peak, negative peak and end. The corresponding NF MUP (*B*) shows the contributions from those fibres closest to the recording electrode as a measure of fibre density.

A NF MUP was taken as the acceleration of a MUP identified by applying a second‐order, low‐pass differentiator to the MUP: this effectively reduces the recording area of the needle electrode to within ∼350 μm, thereby ensuring only the closest fibres significantly contribute to the NF MUP, thus reducing the interference and attenuation artefacts from distant active fibres (Stashuk, 1999 *b*). The NF fibre count was obtained from the template NF MUP and is a measure of fibre density (Fig. 2). NF jiggle is a measure of the variability of consecutive NF MUPs, and was expressed as a percentage of the total template NF MUP area.

### Statistical analysis

MUP parameters and MUNE values from young and old were not normally distributed and were compared using the Mann–Whitney test. CMAPs and sMUPs were compared between young and old using an independent samples *t* test. All correlations were carried out using the Spearman's correlation coefficient (*r*). Data are reported as the mean ± SD or median (interquartile range; IQR), unless stated otherwise. *P* < 0.05 was considered statistically significant.

## Results

Participant characteristics are shown in Table 1. The older participants reported physical activities for health, fitness or leisure purposes >4 h in total over the previous 7 days, and this was representative of their usual activity levels.

**Table 1:** Participant characteristics

	Young (*n* = 22)	Old (*n* = 20)
Age (years)	25.3 ± 4.8	**71.4** ± **6.2** ***
Physical activity level (h per week)	–	6.9 ± 1.7
Height (cm)	177 ± 6	173 ± 7
Weight (kg)	75.8 ± 12.5	75.9 ± 11
BMI (kg/m^2^)	23.9 ± 3.7	25.3 ± 3.7
Body fat (%)	14.8 ± 7.2	**23.8** ± **7.9** **
ALM/h^2^	9.58 ± 1.17	**8.57** ± **0.59** **
SPPB score	12 ± 0.0	11.8 ± 0.4
Grip strength right hand (kg)	50.4 ± 8.6	**40.3** ± **7.1** **
Grip strength left hand (kg)	47.4 ± 6.9	**38.4** ± **7.5** **
Knee extension MVC (*N*)	500 ± 158	**341** ± **109** **
Anterior thigh fat thickness (cm)	0.36 ± 0.16	0.36 ± 0.14
Quadriceps CSA (cm^2^)	89.2 ± 14.9	**59.8** ± **7.5** ***
VL CSA (cm^2^)	29.2 ± 6.2	**18.2** ± **4.3** ***
Femur CSA (cm^2^)	6.28 ± 0.44	6.27 ± 0.67

Data are shown as the mean ± SD. Significant differences between younger and older men are indicated in bold: ^*^ *P* < 0.05, ^**^ *P* < 0.01, ^***^ *P* ≤ 0.0005.

Younger and older participants were similar in height, weight and body mass index. Older participants had a higher total body fat percentage than younger participants and all but one older man achieved full marks in the SPPB. Younger men were stronger than the older men and had larger quadriceps muscles. Anterior thigh fat thickness and femur bone CSA did not differ between younger and older participants.

### MUP characteristics

All participants were able to perform voluntary knee extensions with the concentric needle in position without reporting any adverse reactions.

**Figure 3:**
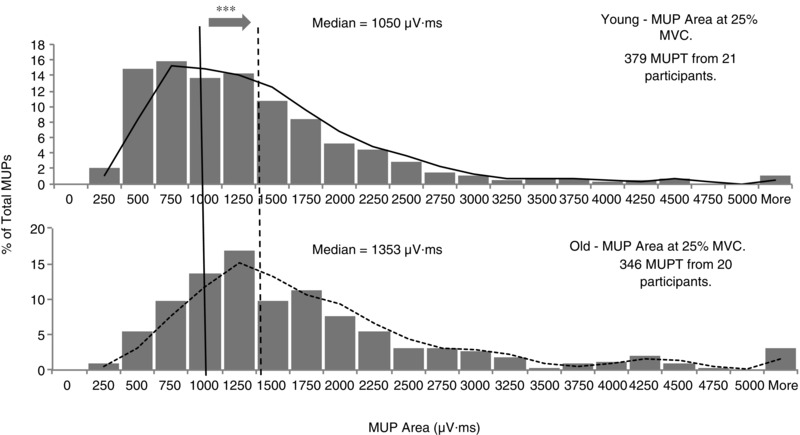
**Frequency distribution of MUP areas detected by iEMG** Data were collected using concentric needle electrodes in younger adults (top) and older adults (bottom) from six spatially distinct regions around the proximal VL motor point during voluntary contractions held at 25% maximal voluntary strength. The vertical lines indicate the median MUP area for younger (solid) and older (dashed) men. ^***^ *P* < 0.0005 indicates a significant difference between younger and older men.

The iEMG data are shown in Table 2. A mean ± SD of 20 ± 5 individual MUs (capturing both MUPs and sMUPs) were sampled per younger man and 17 ± 5 per older man. This gave a total of 379 MUs analysed in the young and 346 in the old. Compared to young MUs, older MUs had larger MUPs with more phases and turns, as well as increased fibre count and NF jiggle, which is consistent with MU remodelling via re‐innervation with nascent NMJs. Figure 3 shows a clear rightward shift in the MUP size distribution in older men compared with young, as they recruited a higher proportion of larger MUs than young in order to generate the same relative force. The discharge rate was slower in old compared to young MUs.

**Table 2:** Motor unit potential characteristics

	Young (*n* = 379 MUs)	Old (*n* = 346 MUs)
Number of phases	2.97 ± 0.94	**3.36** ± **1.13** ***
Number of turns	3.69 ± 1.64	**4.41** ± **2.25** ***
MUP amplitude (μV)	540 (359–856)	**664 (455–1052)** ***
MUP area (μV^ ^ms)	1050 (589–1619)	**1353 (914–1982)** ***
Average intensity (pps)	53.1 ± 19.4	**37.8** ± **2.0** *
NF count	1.66 ± 0.98	**1.97** ± **1.25** ***
NF jiggle (%)	23.7 (19.3–29.5)	**26.2 (4.92–32.5)** ***
NF MUP area (kV s^2^)	2.83 (1.72–5.08)	**3.88 (2.26–6.21)** ***
NF MUP duration (ms)	2.16 (1.56–2.92)	**2.84 (2.12–3.96)** ***
Discharge rate (Hz)	9.56 (8.29–11.3)	**8.81 (7.65–10.49)** ***

MU recordings were collected from six spatially distinct regions around the proximal VL motor point during voluntary contractions held at 25% maximal voluntary strength. Data are shown as the mean ± SD or, if not normally distributed, as the median (IQR). Significant differences between young and old are indicated in bold and: ^*^ *P* < 0.05; ^***^; *P* < 0.0005.

### Spike‐triggered averaging estimation of sMUPs and MUNE

**Figure 4:**
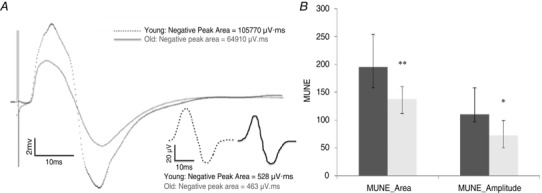
**MUNE values in younger and older men** *A*, example raw traces for CMAP and mean sMUPs (inset). *B*, MUNE values derived from calculations based on area or amplitude of the mean sMUP and the CMAP. Data are the median (IQR). Dashed lines, younger men; continuous lines, older men. ^*^ *P* < 0.05 and ^**^ *P* < 0.01 indicating a significant difference between younger and older men.

Table 3 shows the MUP characteristics collected from sEMG. The CMAP was significantly larger in younger compared to older participants. Although the MUPs were larger in old compared to younger participants (Fig. 3), the representations from surface‐recorded, ensemble‐averaged sMUPs did not differ between young and older participants (Table 3). Figure 4 *A* shows example raw traces for CMAP and mean sMUP potentials. Figure 4 *B* shows the MUNE values in younger and older participants, with the median values being 195 for younger participants and 139 for older participants, when calculated based on area of the CMAP and mean sMUP, and 111 and 78 for younger and older participants, respectively, when calculated based on amplitude. The MUNE values from older men were 71% and 70% of the values from young men when based on area (*P* = 0.008) and amplitude (*P* = 0.018), respectively.

**Table 3:** Surface EMG parameters

	Young (*n* = 22)	Old (*n* = 20)
CMAP area (μV ms)	95125 (20594)	**60371 (21336)** ***
CMAP amplitude (μV)	11260 (2832)	**7690 (2360)** ***
Mean sMUP area (μV ms)	515 ± 251	415 ± 130
Mean sMUP amplitude (μV)	96.7 ± 39.2	87 ± 39.8

Surface EMG recordings were made over the motor point of VL during contractions held at 25% maximal voluntary strength. Data are shown as the mean ± SD. Significant differences between younger and older men are indicated in bold: ^***^ *P* < 0.0005.

There was a strong correlation between all MUNE values derived from area and amplitude (*r* = 0.808; *P* < 0.0005).

The MUNE values were significantly correlated with quadriceps CSA when considering older and younger participants together (*r* = 0.415; *P* = 0.023 when based on MUNE value derived from areas; and *r* = 0.465; *P* = 0.010 when based on MUNE values based on amplitudes), although significance was lost when plotting younger and older participants separately.

## Discussion

The present study aimed to investigate the nature and extent of MU changes in the VL of healthy, independent living, physically active older men. Compared to younger men, older men had fewer MUs and smaller quadriceps muscles. The MUs that remained in the older participants were enlarged, with less stable NMJ transmissions and slowed discharge rates. Consequently, older men recruited larger, and presumably fewer, MUs to maintain the same relative external force as younger men. The extent of MU loss is considerable even in relatively healthy older men and would be expected to have consequences for motor control.

### Participants

The participants recruited for the present study were living independently and exceeded the recommended minimum physical activity levels (DOH, 2011). None of the older participants could be classified as having muscle weakness according to cut‐off values based on grip strength (Dodds *et al*. 2014) and all but one gained maximum marks in the SPPB: a test used to identify clinically significant mobility limitations (Guralnik *et al*. 1994). Furthermore, none of the older men were sarcopenic according to the original definition of muscle loss with ageing (Baumgartner *et al*. 1998) or the more recent version that combines lean mass, grip strength and mobility (Cruz‐Jentoft *et al*. 2010). The lower MU number and function in older men therefore occurred prior to the onset of clinically‐relevant muscle wasting, weakness and mobility impairments.

### Knee extensor size and strength

Even though the older participants were relatively healthy, physically active, non‐obese and similar in stature to young, their quadriceps CSA was 67% that of the young and their MVC was proportionately reduced at 68%. These muscle changes are similar in magnitude to those previously reported in otherwise healthy older men and women (Maden‐Wilkinson *et al*. 2013) of this age and deonstrate the extent of thigh muscle loss and weakness in what would be known as ‘non‐sarcopenic’ older men.

The VL is representative of the general quadriceps atrophy with age (Maden‐Wilkinson *et al*. 2013), comprising a superficial muscle accessible for i.m. and surface EMG, and the femoral nerve can be easily stimulated to record a CMAP. The VL is a muscle that is frequently biopsied, such that the fibre morphology and physiology have been studied extensively, and it has a large CSA that minimizes EMG cross‐talk from nearby muscles.

### MUPs

The iEMG recordings in the older men showed higher values for MUP size (area, amplitude), complexity (number of phases, number of turns) and fibre density (NF count), along with slower discharge rates, compared to younger men (Table 2). The VL MUP amplitudes and areas were 23% and 29% larger in older compared to younger men, respectively. Area and amplitude of the MUPs correlated strongly (*r* = 0.871; *P* < 0.0005).

Larger MUPs are generally taken to indicate MUs with a greater number of muscle fibres, giving a greater innervation ratio, and this is consistent with the increased NF count in older participants (Table 2). The process of collateral re‐innervation of orphaned fibres that increases the number of fibres per MU as a compensatory mechanism (McComas *et al*. 1993; Luff, 1998) serves to preserve total muscle force generating capacity against the backdrop of motor neuron losses. Re‐innervation is not completely successful, however, because ∼40% of fibres in VL are lost by ∼75 years old (Lexell *et al*. 1988), although our own estimates based on unpublished data for differences in muscle fibre and quadriceps size suggest a figure closer to 20% loss in healthy ageing. Other changes possibly also affecting MU function in the old are increased NF jiggle, number of turns and phases (Table 2), indicating discharge variability and asynchronous action potential transmission within the same MU (Nandedkar *et al*. 1988).

The MU discharge rates were, on average, ∼10 Hz in younger participants and 9 Hz in older participants, with ranges of 5–18 Hz and 5–15 Hz in the younger and older participants, respectively. This modest reduction in the older participants is similar to reports of tibialis anterior (Connelly *et al*. 1999; Patten *et al*. 2001; Klass *et al*. 2008) and first dorsal interosseous (FDI) (Kamen *et al*. 1995), although a previous study in VM showed no difference between young and old MU discharge rates (Roos *et al*. 1999). The slightly lower discharge rate in the older participants may be related to altered central drive (Klass *et al*. 2008), although it is probable that the older participants achieved the required external force (25% MVC) by recruiting fewer MUs (i.e. a greater reliance on early recruited MUs that have lower firing rates than higher threshold MUs) (Conwit *et al*. 1999), which were contributing a higher force per MU compared to the young (Ling *et al*. 2009).

The finding of larger MUPs in healthy older people was first highlighted in the extensor digitorum brevis by Campbell *et al*. (1973) and has subsequently been shown across a range of limb muscles. The technique of incremental nerve stimulation has been used to reveal higher MUP area in older compared to younger individuals in the extensor digitorum brevis and thenar muscles (Galea, 1996). The MUP amplitude was higher in older compared to younger soleus across a range of voluntary contraction intensities (Dalton *et al*. 2008). More recently, the MUP area was found to be higher in the VM of older compared to younger individuals when holding a voluntary contraction matched for ‘average pps intensity’ (Hourigan *et al*. 2015). Matching of the pps may not be a good way of comparing younger and older individuals because we found pps to be lower in older compared to younger individuals when working at the same relative external force (Table 2). The increased NF jiggle in older men reported by Hourigan *et al*. (2015) was similar to the values reported in the present study for VL muscle (Table 2). The higher NF jiggle is taken to indicate variability in muscle fibre potential transmission along fibres within the MU possibly as a result of the increased transmission variability of nascent NMJs of newly reinnervated fibres (Hourigan *et al*. (2015).

By contrast to the present observations, Galea (1996) found the MUP area to be ∼25% smaller in older biceps brachii, which may indicate that motor axon ‘sprouting’ could be less successful in the biceps brachii. However, studies of age‐related changes to MUs in the biceps brachii have provided mixed results (Brown *et al*. 1988; Doherty *et al*. 1993; McComas *et al*. 1993; Power *et al*. 2012).

### MU number estimation

The VL MUNE values were positively associated with VL muscle size, indicating that a loss of motor neurons contributes to loss of muscle mass with ageing, although the significance of this relationship was lost when limiting the correlation to only the smaller group of older men. Previous studies did not report muscle size in relation to MUNE values, although Kaya *et al*. (2013) used a non‐invasive index of MU numbers to show that low values were associated with muscle weakness.

The MUNE values depend on two measurements: the area or amplitude of the surface recorded CMAP and the area or amplitude of a mean sMUP. On average, the sMUPs of the older participants were 81% of those for the younger participants, whereas the older CMAPs were 68% of those for the younger CMAPS. Area‐based MUNE values for the older participants were 71% of those for the younger participants, whereas the amplitude‐based MUNE values were 70% of those for the younger participants (Fig. 4). Although MUNE values are a widely used representation of MU numbers, there are aspects of this methodology that require some comment, particularly relating to the CMAP.

The CMAP is obtained by maximally stimulating the motor nerve and the expectation is that all MUs in the muscle will have been activated and thus the CMAP is the summation of the electrical activity of all the MUs in the muscle. This depends, however, on the volume of muscle ‘seen’ by the recoding electrodes. For a small muscle such as the FDI or thenar, the surface electrode's recording area will probably encompass the whole muscle. Several studies have been based on the assumption that the CMAP is related to the total number of axons, and thus presumably MUs, within the muscle (Kurokawa *et al*. 1999; Wee, 2006; Severinsen & Andersen, 2007), although the occurrence of cross‐contamination from other muscles may be a problem. For a large muscle such as the VL, the CMAP probably does not represent the firing of all units. The MUNE values that we find for the VL (Fig. 4) are similar to those reported for other smaller muscles (Galea, 1996; McNeil *et al*. 2005; Dalton *et al*. 2008; Power *et al*. 2010; Power *et al*. 2012). This suggests that the MUNE values reported in the present study for the VL are an estimate of the number of MUs within a volume of muscle rather than the whole muscle.

Barkhaus & Nandedkar (1994) estimate that the CMAP is recoded from a depth of ∼2 cm, which is a small proportion of the total VL in younger and older participants. The CMAP recorded from the older participants was consistently smaller than that from the younger participants, an observation also reported previously (Dalton *et al*. 2008; Power *et al*. 2012). The lower CMAP in the older participants is probably not a result of fibre atrophy alone because two studies of disuse atrophy in younger participants showed that CMAP amplitude did not change in the soleus (Clark *et al*. 2006) or the FDI (Fuglevand *et al*. 1995). The reduced CMAP in the older participants may be the result of relatively fewer muscle fibres within the recording area of the electrodes, as well as interference effects of i.m. fat and connective tissue, which increase in healthy aged muscle (Lexell *et al*. 1988; Hogrel *et al*. 2015), causing diminution of signals recorded at the surface.

The same factors causing attenuation of the CMAP will also affect the sMUPs because both were recorded by the same sEMG electrode. The sMUPs were identified after being ‘triggered’ from the i.m.‐recorded MUPs (Brown *et al*. 1988; Stashuk, 1999 *a*) and, despite the MUPs being larger in older than younger participants, the sMUPs were (non‐significantly) smaller in the older participants, which was previously reported in studies of MUNE (Dalton *et al*. 2008). Whatever the cause of attenuation of the CMAPs and sMUPs of the younger and older participants, we can expect that, when recording from a specific muscle (of a younger or older participant), the CMAP and sMUP will be similarly affected by the electro‐physiological characteristics of the muscle and overlying tissue.

If MUNE values represent the number of MUs in the same CSA of muscle in younger and older individuals, it is possible to estimate differences with respect to the total number of MUs in the whole muscle, knowing the differences in CSA of the VL. From Table 1, the CSAs are 29.2 and 18.2 cm^2^, respectively. In this case, the total number of MUs will be proportional to the MUNE value multiplied by CSA (5694 and 2530 for younger and older individuals, respectively, based on MUNE area, and 3241 and 1420, based on amplitude). Thus, the total number of MUs in the VL of the older participants is either 44% or 42% of the value for younger individuals depending on whether area or amplitude based MUNE values are considered.

There is an alternative approach for estimating the effects of age on MU number that does not depend on any assumption about the CSA of the muscle represented by a MUNE value. This alternative makes only the assumption that MUP area or amplitude is indicative of the size of the MU that generated the MUP. Thus, dividing VL muscle CSA by mean MUP area provides a value that is proportional to the total number of MUs in the VL muscle. This gives values of 0.026 and 0.012 for younger and older individuals, respectively (the units being cm^2^ divided by μV ms), with the value for older individuals being 46% of the value for younger individuals. Based on MUP amplitude, older individuals have 48% of the MU number compared to younger individuals. These conclusions are remarkably similar to the older participants having 44% and 42% of the younger MUNE values derived from the CMAP and mean sMUP described above. These values are somewhat greater than the ∼30% motor neuron loss estimated from cadaveric specimens (Tomlinson & Irving, 1977).

Therefore, it is evident that the major change in older muscle is a loss of MUs that is partly offset by re‐innervation of denervated fibres and enlargement of surviving MUs. Fibre atrophy plays a lesser role in the age‐related loss of the VL muscle, a conclusion that is also reached from an examination of cadaveric specimens (Tomlinson & Irving, 1977).

### Limitations

The values that we report in the present study are based on MUs activated at 25% of MVC and recorded from the mid‐muscle belly. Based on the size order of recruitment (Henneman *et al*. 1965), it is possible that we are sampling from the smaller and medium MUs within the entire pool. With an increase in contraction level, we would expect an increase in MU size, reflected in the mean sMUP, which, once divided into the CMAP, would result in a smaller MUNE. In practice, however, it is more difficult to extract MUPTs created by individual larger MUs during high force voluntary contractions.

A loss of motor neurons and the associated remodelling of surviving MUs places restrictions on the central nervous system in terms of programmed motor pathways. This will most probably affect motor control in old age but, to date, the effects of MU remodelling on the control of movements remains largely unknown.

### Conclusions

The present study has described, for the first time, MU remodelling in the VL during healthy ageing in terms of a reduction in the total number of MUs; an increase in the size of the surviving MUs; an increased instability in NMJ transmission; and a decrease in surviving MU firing rates. These changes have a significant impact on overall muscle loss. Further studies are required to clarify the effects of this MU remodelling on muscle function and mobility.

## Additional information

### Competing interests

The authors declare that they have no competing interests.

### Author contributions

JM was the principle investigator of the research project. MP, AI, DJ and JM performed the experiments, analysed the data and drafted the manuscript. DS and AHW provided decomposition analysis software and assisted in the data analysis. Experiments were conducted at the School of Healthcare Science, Manchester Metropolitan University. All authors have approved the final version of the manuscript and agree to be accountable for all aspects of the work. All persons designated as authors qualify for authorship, and all those who qualify for authorship are listed.

### Funding

This work was supported by funding from UK Medical Research Council (MR/K025252/1).
